# How Do Nursing Students Perceive Care Towards the LGBTIQ+ Community? A Phenomenological Study on Sexual and Gender Diversity

**DOI:** 10.3390/nursrep15050155

**Published:** 2025-04-30

**Authors:** Carlos Daniel Lemus Celin, Candy Laurine Suarez-Albor, Felice Curcio, Dhurata Ivziku, Olga Esther Hernandez Almanza, Mauro Giovanni Carta, Cesar Iván Avilés González

**Affiliations:** 1 Department of Nursing, Universidad Popular del Cesar, Valledupar 200002, Colombia; cdlemus@unicesar.edu.co (C.D.L.C.); vidasaludable@valledupar-cesar.gov.co (C.L.S.-A.); olgahernandez@unicesar.edu.co (O.E.H.A.); mauro.carta@unica.it (M.G.C.); 2 Local Health Secretary of the Municipality of Valledupar, Valledupar 200002, Colombia; 3 Faculty of Medicine and Surgery, University of Sassari (UNISS), Viale San Pietro 43/B, 07100 Sassari, Italy; 4 Department of Health Professions, Fondazione Policlinico Universitario Campus Bio-Medico, 00128 Rome, Italy; d.ivziku@policlinicocampus.it; 5 Hospital Rosario Pumarejo de López, Valledupar 200002, Colombia; 6 Department of Medical Sciences and Public Health, University of Cagliari, 09042 Cagliari, Italy; 7 Department of Nursing, University of Studies of Enna Kore, 94100 Enna, Italy; cesar.aviles@unikore.it

**Keywords:** attitudes, gender minority, health services, LGBTIQ, nursing care, student, nursing, social, stigma

## Abstract

**Background/Objectives**: Care-oriented perspectives in the LGBTIQ+ community are essential to ensure adequate and comprehensive health care. This population faces multiple barriers imposed by society, including discrimination in access to healthcare services. This research aims to analyse the perspective of nursing students in relation to the provision of care for people with sexual and gender diversity. **Methods**: A qualitative phenomenological research-action study was conducted. Data were collected using a semi-structured face-to-face interview consisting of 23 questions. The interviews were transcribed, read thoroughly and analysed. **Results**: Forty students were interviewed, and four main themes emerged: (1) personal perspective, (2) professional and disciplinary training, (3) nursing education and (4) health implications. The results show that students have mixed perspectives and attitudes towards LGBTQ+ populations, although they are interested in receiving more LGBTQ-focused training. Furthermore, respondents reported that the training received in the degree course and the preparation of lecturers in relation to caring for LGBTQ+ people are lacking. Finally, it emerged that inequalities in access to health services can still be observed and that there is a need for the development of manuals that are at the forefront of comprehensive health in this population. **Conclusions**: Based on the results, the implementation of Madeleine Leininger’s Sunrise model is suggested. Acceptance of sexual and gender diversity is essential to ensure the elimination of inequalities and make care a transformative process. Finally, education on these aspects needs to be implemented by including activities such as practical training scenarios, workshops and conferences to highlight the specific needs of the LGBTIQ+ population.

## 1. Introduction

Sexual and gender diversity refers to the different alternatives that individuals have to express, feel and accept their sexuality, to assign themselves an identity or orientation according to their personality [1,2]. Lesbian, gay, bisexual, transgender, intersex and queer people and people with a diversity of sexual and gender identities (LGBTIQ+) are often marginalised and suffer discrimination in accessing health services [3,4,5].

Globally, the gap between the growing scientific knowledge of the health needs of LGBTIQ+ people and the evolution of healthcare for this population is well known. Indeed, a considerable percentage of LGBTIQ+ people still face stigmas, prejudice, discrimination and even violence, which often take place within health services [6,7,8,9]. The World Health Organisation (WHO) and the Pan American Health Organisation (PAHO) have highlighted the need to address health inequalities that make the LGBTIQ+ community vulnerable, pointing out that discrimination and stigma are barriers to accessing health services [10,11,12]. Therefore, differentially oriented health care is a fundamental aspect of the well-being of gender-diverse individuals [13]. Especially at a time where sexual and gender diversity has been recognised and has acquired equal rights [14,15], there is a need to strengthen the knowledge and skills of students and health professionals so that they are trained to face these challenges and eradicate social inequalities [16,17,18].

Internationally, studies show that a lack of education on sexual diversity issues may contribute to discriminatory behaviour and inadequate and outdated care spaces [19,20,21,22].

The study conducted by de Lima e Silva and Travassos [23] suggests that students trained with an inclusive view of sexual diversity develop empathic, comprehensive and therapeutic skills towards LGBTIQ+ patients, resulting in competent and quality care. Furthermore, it emphasises that the preparedness deficit associated with the LGBTIQ+ population predisposes them to discriminatory and stigmatising clinical environments. Similarly, the Amorim Costa et al. [24] study suggests that nursing students frequently experience insecurities when interacting with LGBTIQ+ people, leading to ineffective therapeutic measures. This barrier can be attributed to poor training in academic programmes, highlighting the need to update curricula and strengthen thematic content in these areas of knowledge [16,25,26]. The implementation of practical training, through simulation workshops, workshops focusing on sexual diversity, the use of LGBTIQ+ cultural competence programmes or documentaries about their experiences, can improve the knowledge, attitudes, self-efficacy, confidence and intentions of nursing students and other healthcare professionals in providing culturally competent care to LGBTIQ+ patients, eliminating disparaging judgements and creating meaningful experiences for these patients [27,28,29,30,31]. From a nursing perspective, where professional practice is oriented towards the philosophy of care, it is essential to adhere to the holistic perspective [32], which integrates biological and social aspects. Madeleine Leininger’s Sunrise model emphasises culture and caring as essential concepts in nursing care and, considering the multiplicity of different cultural aspects and the specific needs of each social group, helps nurses to be respectful and understanding of diversity [33,34,35].

The adoption of diversity-friendly care is not only ethically and legally indispensable, but also has a positive impact on people’s well-being [36,37]. The creation of an equitable health care system free of prejudicial barriers would facilitate and increase access to health services, such as prevention, diagnostic support, treatment adherence and improved quality of life in the LGBTIQ+ population [38,39,40]. Providing culturally diverse nursing care is a major challenge today, and this is reflected in the importance of strengthening health, both mental and physical, in the LGBTIQ+ population by reducing inequalities, prejudice and misunderstanding [5,41,42].

This research contributes significantly to the nursing discipline by highlighting the key role of education and training of nursing students in promoting inclusive care for the LGBTIQ+ population. The results highlight the need to integrate specific modules on sexual and gender diversity into academic curricula to improve cultural competence and reduce health disparities. Furthermore, the application of Madeleine Leininger’s transcultural theory emerges as an essential theoretical framework to provide holistic, respectful and non-biased nursing care. The implementation of educational strategies based on simulations, clinical scenarios and awareness-raising programmes can foster a more equitable and accessible healthcare environment for all patients, regardless of their gender identity or sexual orientation. In this sense, the practical implications of this study suggest the need for healthcare policies that promote a differentiated and diversity-friendly approach to care, thus ensuring a better quality of nursing care for the LGBTIQ+ community.

In Colombia, the situation of people with sexual and gender diversity has seen significant progress in recent decades but still remains complex, especially in relation to cultural and social issues. Colombia is a nation with a history of discrimination against LGBTIQ+ people, but has also made significant steps towards inclusion and civil rights, such as the legalisation of egalitarian marriage, recognition of the right to legally change one’s gender, etc. Despite legal progress, access to inclusive healthcare remains a critical issue [43]. A 2008 report provided by the Bogota mayor’s office found that LGBTIQ+ patients who reported their sexual orientation and gender identity to health professionals were more likely to be discriminated against in healthcare services and treatment. The report revealed a lack of culturally sensitive health education for staff providing health services to people with sexual and gender diversity [44].

The aim of this study was to analyse the perspective of nursing students regarding the provision of care to people with sexual and gender diversity.

## 2. Materials and Methods

### 2.1. Design

Through action research, researchers identify a problem in their context and attempt to study it in order to understand, solve and/or improve it [45]. This study employs a qualitative research design using Interpretative Phenomenological Analysis (I.P.A.), a qualitative research approach that values ‘a detailed experiential account of the person’s involvement in the context’ [46]. This design aims to explore nursing students’ experiences of sexual and gender diversity.

The IPA framework allows for an understanding of the meanings of communication through the narration of participants’ experiences from cultural, social and personal perspectives [47,48]. In line with Heidegger’s concept, according to which the experiences of individuals are intrinsically shaped by the world around them, Interpretative Phenomenological Analysis recognises that meanings are always generated through interactions with other subjects. This mix of qualitative research methods allows for an in-depth and detailed exploration of the emotional, practical and relational impacts of the LGBTIQ+ population on nursing students.

The results of this study are reported in accordance with the Consolidated Criteria for Reporting Qualitative Research (COREQ) [49].

### 2.2. Sampling and Recruitment

Between 8 May and 30 June 2024, all nursing students at the Universidad Popular del Cesar (Colombia), who were completing a professional internship, were invited by the research team to participate in the study.

We employed an intentional sampling strategy to generate a heterogeneous sample of students in terms of the semester they were attending. The sampling aimed to maximise the variation between the different clinical contexts (public health, primary care, mental health, medical–surgical, maternal–child, health education, internal medicine and health management). This intentional sampling, conducted by the CDLC author, ensured that we captured the different perspectives and experiences of each student and how they interfere with the quality of care provided to an LGBTIQ+ patient. The recruitment of participants continued until we reached information saturation, defined as the point at which no further themes and sub-themes emerged from the data [48]. This sample size was considered adequate to collect rich and comprehensive data that captures the unique perspective of each participant.

This approach allowed for an in-depth exploration of participants’ lived experiences, in line with the principles of Interpretive Phenomenological Analysis (IPA), which allows us to understand how people perceive their experiences in specific settings.

### 2.3. Data Collection

Participants were invited via institutional e-mail. Only the researchers and the interviewee were present during the interviews. None declined the interview. Prior to data collection, each researcher involved in the study performed bracketing, noting down ideas, preconceptions, beliefs and convictions about the phenomenon under investigation [50]. This step is crucial because researchers’ preconceived notions could affect data analysis in studies using a phenomenological interpretive approach. By applying this ‘reflexive technique’ before data collection and analysis, researchers can focus greater attention on avoiding introducing preconceptions and/or prejudices that could negatively influence the research [51].

Data were collected via semi-structured, face-to-face interviews. This type of interview, which is the most frequent method of data collection in IPA research, was chosen because it is particularly informative, allowing the researcher to create a logical progression for the topics addressed. Although characterised by the fact that the same questions are asked of the interviewees, even with the same wording and in the same order, it is the interviewee’s answers that determine the direction of the interviews [52]. In addition, the semi-structured interview guide offers a clear set of instructions for interviewers and, at the same time, can provide reliable and comparable qualitative data.

The interviews initially collected some demographic information, including age, gender and sexual preference. This introductory phase contributed to contextualising the participants’ answers and provided background for a better understanding of the different perspectives. An interview guide (Appendix A) was used flexibly to encourage participants to process their perceptions through natural conversation, on the one hand, and to provide clear instructions for interviewers on the other. In addition, field notes were collected to capture non-verbal signals, tone of voice and emotional reactions that were not easily detectable in the audio recordings, and that enriched the understanding of the experiences studied. The first question, ‘What is your perception of LGBTIQ+ people?’, was used to introduce the topic. Each interview lasted an average of 20 min; no minimum duration was set by default, as the interviewer let the participants express themselves in complete freedom.

The pilot interview was submitted to three nursing students, different from the participants, who did not suggest changes to the questions, suggesting that they were clear and concise. In addition, the principal investigator was trained to conduct sensitive discussions by attending a training course on differentiated health care for LGBTI people held by the district centre for health education and research of the health secretariat-mayor’s office in Bogotá, Colombia.

### 2.4. Data Analysis

The interviews were recorded in audio format and archived on the principal investigator’s computer, which was password protected. Each interview was coded with initials corresponding to the context of the clinical internship and a numeric character to maintain the consecutive order of each interviewee, e.g., the first interviewee attending the public health internship was coded as SP_1, the second attending the maternal and child health internship as MI_2, etc.

Data analysis was conducted following the IPA principles suggested by Smith et al. [48]. Initially, all recordings were listened to several times and transcribed in full. Each transcript was read and re-read several times to enable the researchers to familiarise themselves with the content of the interviews. Subsequently, each transcript was analysed line by line, noting descriptive, linguistic and conceptual aspects in the left margin. Reflecting both the transcript and the initial annotations, emerging themes for each interview were reported in the right margin. As IPA follows an idiographic approach, each transcript was considered individually to identify themes before moving on to the next transcript. Subsequently, the emerging themes were connected to establish a coherent and organised thematic account of the phenomenon. Multiple linkages were established, and a set of superordinate themes was developed for the entire corpus of data. Each superordinate theme was linked to the underlying themes, which in turn were linked to the original transcripts of the interviewees.

The triangulation of information was carried out through primary sources derived from the results of the semi-structured interviews, as well as secondary sources, such as similar national and international research. In addition, the themes were independently reviewed by three researchers (C.D.L.C., C.L.S-A. and O.E.H.A.) to ensure that they were grounded and well represented in the transcripts.

Finally, a reflective diary was kept, in which prejudices, personal experiences and preconceptions were discussed within the research team. In Interpretative Phenomenological Analysis, transparency and reflection on researchers’ opinions, experiences and preconceptions is crucial, as is in-depth reflection on how these influences may impact on the interpretation of data and themes [53].

### 2.5. Rigor

The rigour of this study was achieved by applying the criteria of reliability, credibility, confirmability and transferability recommended by Streubert and Carpenter [54]. The data were transcribed as direct citations from the interviews and interpreted using the thematic approach technique, safeguarding the fidelity of the interpretations from the possible judgements of the interviewees. Inclusion/exclusion criteria, context, respondent characteristics, data collection and analysis procedures were detailed [55]. To minimise the risk of confirmation bias, the results were shared between the researchers and with the participants. This rigour ensures the validity, pertinence and relevance of the results obtained, contributing to a comprehensive understanding of care with a differential approach in the training process of future health professionals.

### 2.6. Ethical Considerations

This project was approved by the Universidad Popular del Cesar (Cesar, Colombia), which was included in the internal call for young graduate researchers 2023, developed under an institutional agreement valid from February to December 2024.

The study was carried out in accordance with the Declaration of Helsinki [54], the provisions of the Colombian data protection law [55] and Agreement No. 019 of 5 October 2020, the data-processing policy of the Universidad Popular del Cesar, Colombia [56]. The interviews were authorised by the Vice-Rectorate for Institutional Research (Implementation Agreement No. 098 of 22 December 2023).

All participants were thoroughly informed, both orally and in writing, about the purpose of the study. Prior to the interviews, each participant signed an informed consent form, stating that they understood the purpose of the study, that they participated voluntarily, that they had the right to withdraw at any time and that this choice would not affect their university career. In addition, along with the informed consent, a document called a confidentiality letter was provided, where it was assured that the anonymity and confidentiality of the data would be guaranteed by the CDLC author, being coded and kept anonymous without the possibility of identifying the participants.

## 3. Results

Forty students from the Faculty of Health Science, nursing programme of the Universidad Popular del Cesar participated in this research. They were carrying out training practices at the Rosario Pumarejo de López hospital, Valledupar, Colombia. The majority of the sample (67.5%, n = 27) were female students, while male students represented 32.5% (n = 13). With regard to sexual preference, 82.5% (n = 33) of the students stated that they had a heterosexual orientation, 7.5% (n = 3) stated that they considered themselves gay, 7.5% (n = 3) bisexual and, finally, the remaining 2.5% (n = 1) stated that they were non-binary. As can be seen, the majority of the study population leads a sexual and emotional life with the opposite sex, while 17.5% (n = 7) of the future nurses belong to the LGBTIQ+ community. In relation to age, the respondents had an average age of 21, ranging from 18 to 26 years.

The analysis of the data collected on the nursing perspective identified four main themes (Table 1).

### 3.1. Personal Perspective

In order to understand nursing students’ personal perspectives on nursing, questions were formulated to identify the respondents’ opinions, thoughts, feelings and experiences with regard to sexual and gender diversity. It is important to emphasise that when investigating the personal perspective, it is possible to find different views, opinions and ideologies. In fact, three different positions can be observed from the interviews conducted.

Firstly, favourable views of the LGBTIQ+ population are observed. One group of students, in fact, states that ‘they have the right to care without stigma or barriers’ (E_ES1), stating that an individual’s sexuality is not a factor in defining them as different from others: ‘I have the perception that they are normal people, with difficulties and virtues like all people; I also believe that belonging to this is not a condition of a disability (…)’ (E_SM2). Furthermore, the nursing students underline the Colombian state’s legislative commitment, which, through social policies, guarantees that the LGBTIQ+ population cannot be disturbed and/or mistreated in society. E_GS4 reports: ‘I would start by saying that we are all human beings (…); we all have differences, but on the assumption that, thanks to the political constitution of 1991, in Colombia we are individuals with rights, and we are free to express ourselves, respecting the rights of others’. The above allows us to observe that future nursing professionals do not make distinctions between people based on their tastes, choices or sexual orientation.

Secondly, a group of interviewees has a negative perception of the LGBTIQ+ population. This group of students, through expressions, gestures and non-verbal language, reflects feelings and/or attitudes of rejection towards this community. Prominent among the various perceptions is that of those who believe that people of different sexes are ‘empty and seeking approval from others’ (E_MI4). Similarly, some interviewees refer to experiences in which the behaviour of some people in the LGBTIQ+ community appeared inappropriate; hence the negative perception towards them. E_MICC reports that ‘there are things he does not agree with, because they are not in accordance with what society requires; there are gays who want to be women and denigrate the biological woman a lot’. The above comments reflect the existing belief, even today; having sexual diversity brings out a differential line in the social aspect that can lead to the consolidation of rejection in various environments, including the health sector.

Finally, neutral perspectives on the LGBTIQ+ population emerge from the interviews collected. Some nursing students report that they have no feelings towards this population, ‘I don’t reject them, but I don’t support them either’ (E_MI2). Students who express neutral attitudes might reflect the influence of implicit prejudices. These biases, being unconscious, may influence perceptions and behaviour towards sexual and gender diversity. Several studies emphasise the importance of diversity training that enables future professionals to recognise and manage these biases, thus promoting more inclusive environments. This neutral perspective does not make it possible to understand whether it is beneficial or not when exercising the role of caregiver in the LGBTIQ+ population. However, a relevant aspect of nursing students’ perspective of care is related to the social acceptance of the LGBTIQ+ population. This makes it possible to provide comprehensive, effective and quality care.

A significant proportion of the interviewees claim that taking to the streets, marching, is synonymous with raising one’s voice and creating an echo, making oneself visible and claiming rights denied to them by heteronormative social behaviour. E_MI5 reports ‘Unfortunately, every year they are forced to take to the streets to be seen, to be noticed because they are considered a minority (…). I think it is very brave of them …, they are not hurting anyone, they are simply asserting their rights through demonstrations. Nothing wrong with that!’. On the other hand, a group of interviewees claim that it is a way to attract attention, resulting in ridicule and social prejudice. Furthermore, they claim that the demonstrations they organise are vulgar, obscene and immoral—‘they attract attention in order to be ridiculed’ (E_ES5). Similarly, a small group of participants declare that they maintain a neutral stance in the face of these types of demonstrations, expressing that they do not have any feelings for or against them.

When students’ perceptions of violence towards people with sexual diversity were investigated, the majority of the participants stated that they did not support any type of violence. E_ ES3 reports: ‘they are totally against being criticised, physically, verbally and emotionally attacked just because of their sexual preferences’. Furthermore, it is made clear that such acts of violence are due to behavioural problems of the aggressors: ’there is resentment that drives the aggressor to such actions’ (E_ ES4). On the other hand, a small group of interviewees report feelings of non-acceptance towards the LGBTIQ+ population, supporting and justifying acts of aggression as if they were deserved actions for their choices’ (E_ES5).

An important aspect to consider is the influence of religion on the LGBTIQ+ population. Religion can both facilitate and hinder each participant’s opinion, although most respondents report that their beliefs are personally and professionally empowering, commenting that ‘religion teaches one to see every person as a decent being, regardless of their sexual orientation’ (E_CB1). However, some interviewees point to conflicting situations where doctrine and reason do not overlap: ‘I feel that my beliefs clash with what I learn about diversity. It is difficult to find a balance’ (E_GS4).

### 3.2. Professional and Disciplinary Training

The nursing students interviewed state that their academic training in relation to sexual diversity was deficient and sporadic. E_ES1 states that ’the university is still a bit conservative.’ Similarly, some respondents state that topics on sexual diversity are addressed in a hasty manner, as stated by E_MI3: ’topics on diversity are rarely addressed.’ In general, students state that this topic is not addressed in any subject; a small group states that teaching in the maternal–child area does address diversity, but mostly with a cultural focus; likewise, some respondents state that teaching in mental health and psychiatry provides tools for differentiated health care. Finally, one participant states that all nursing teaching provides thematic content for the development of competencies for differential care.

On the other hand, opinions arise that teachers lack adequate skills in diversity, ‘many of my teachers do not know what the acronym LGBTIQ+ means’ (E_GS1). When evaluating the degree of knowledge about the differential approach, it is observed that the singularity of this approach predominates in the sample of students, which is defined as ‘giving individualized attention to a group with particular characteristics and doing so in a comprehensive way’ (E_MICC4). However, there is evidence that some are unaware of the purpose of the approach, as evidenced by statements such as ’a different approach’ (E_MICC2).

Inequalities in access to health services are conditions that can still be observed today, reaffirming this position through experiences lived by participants, such as ’I once observed that a lesbian girl was not being treated because, apparently, she was not the same person as the one on her identity card’ (E_SM5). Students also point out the interposition of obstacles to the care of sexually diverse people, observed by the ‘inadequate and poor treatment of professionals, which leads to tense and non-therapeutic environments’ (E_MI3). On the other hand, the rest of the respondents state that they do not expect abusive behaviour from this population; many believe that ‘this is a thing of the past and that they do not believe that such behaviours currently exist’ (E_GS1).

It was possible to identify that some participants consider that the most serious health problem in this population is probably ’sexually transmitted diseases’ (E_MICC1). On the other hand, they allow themselves to mention mental disorders such as ’depression, suicidal behaviour, anxiety and isolation’ (E_MQ4). Finally, they relate the problem to the indiscriminate use of hormones and unreliable surgeries manifested in expressions such as ’the inappropriate use of transitional therapies’ (E_MI5).

Although the depathologization of different sexual orientations has been achieved for some time, it is relevant to state that a small group of participants considered that ’there is some problem in LGBTIQ+ people, perhaps childhood traumas’ (E_MQ1). On the other hand, it was revealed that more than half of the respondents were not aware of the LGBTIQ+ acronym, stating that they ’did not remember it at the moment’ (E_CB5).

### 3.3. Nursing Education

Unanimously, students stated that they had no prior knowledge of guidelines or training tools for caring for sexually diverse populations, which demonstrates a great need in the professional future. Most of the respondents consider it relevant to create a manual that is at the forefront of global health in this type of population, highlighting that ’it is a significant advance for global health led by nurses’ (E_SM2), while there are those who affirm that ’nursing should not have gender, that is why there is no need for a manual’ (E_GS4).

Although the Universidad Popular del Cesar has implemented equity and non-discrimination policies, when evaluating their social dissemination, it appears that a third of the students ‘did not know that this policy existed’ (E_SP4). Some of those who are aware of it say they know about it thanks to communication channels external to the nursing program; for example, E_GS4 says ‘they learned about this policy by attending a wellness event organized by the psychology program’. Finally, some of the respondents learned about the equity and non-discrimination policies through the university’s Forums and website, but not through real socialization channels: ‘the university has a Facebook group, there the student representative shared it and informed me’ (E_CB1).

### 3.4. Health Implications

Inequalities in access to health services for gender diverse communities may not only negatively reflect on the physical state but also on the mental health of this population. From the interviews conducted, a number of terms emerged, reported by the participants, that emphasise situations that may jeopardise emotional stability and, consequently, contribute in some way to health deterioration. Among the various terms that emerged, we have

- Discrimination
  The majority of respondents consider discrimination as a catalyst in the physical and mental condition of LGBTIQ+ people. In this regard, E_MICC5 reports that ‘discrimination contributes to rejection, and many people have committed suicide because of it’. Even today, behaviours of discrimination and repulsion against sexually diverse people are present in our society. These attitudes can be observed in a variety of environments, including in the family, academia, work and health services. Such discrimination can increase the risk of suffering from mental disorders such as depression, anxiety, post-traumatic stress and other related conditions. Furthermore, discrimination can exacerbate barriers to access to health services, which in turn affect health promotion and maintenance services
- Violation of rights
  Similarly, interviewed nursing students believe that unequal rights can have major consequences for the integrity of individuals. E_ES2 reports that ‘the violation of human rights in the LGBTIQ+ community results in devastating impacts on the general well-being. When discrimination, rejection or marginalisation of social dissidents is practised, it generates devastating consequences for each individual’.
- Bullying
  In general, interviewees mention that bullying is a lethal weapon that exacerbates conditions for members of the LGBTIQ+ population. ‘Bullying is one of the most romantic ways to mistreat these types of people, unfortunately few report it or have the support to put an end to this psychological phenomenon’ (E_SM3).
- Stereotypes
  Finally, with regard to attitudes that can influence the LGBTIQ+ community, stereotypes emerge. Participants agree on their impact on the stability of the community: ‘all these stereotypical thoughts build rejection behaviours, which in turn hinder holistic care’ (E_SP1). Furthermore, they report that these attitudes in healthcare environments can diminish user confidence, in turn interposing stigmatising barriers that limit appropriate access to the healthcare system.

## 4. Discussion

This study aimed to explore the perspective of nursing students in relation to caring for people with sexual and gender diversity. Overall, the students interviewed showed mixed attitudes towards LGBTQ+ populations, although they were interested in receiving more LGBTQ-focused training. Furthermore, the interviewees reported that the training received in the degree course and the preparation of lecturers in relation to caring for LGBTQ+ people to date are not entirely sufficient.

The results of this study connect to some research, conducted in various contexts, that emphasises the importance of strengthening care for populations with sexual and gender diversity. Through structural changes combined with increased knowledge and understanding of the gaps in health and health care knowledge for the LGBTI population, services can potentially become more inclusive and equally accessible to all [56]. Forrisi, in his study conducted in Uruguay, states that university education on sexual diversity not only strengthens the cultural aspect but also promotes a therapeutic environment in the nurse–patient relationship [35]. Similarly, Arenas [57] suggests that professionals’ lack of preparation leads to negative experiences for LGBTIQ+ patients. In line with our findings, several studies indicate that students report inadequate education on certain topics, with limitations in their knowledge and preparedness to care for LGBTQ+ patients, particularly transgender and gender diverse patients [18]. Neutral attitudes, even if seemingly impartial, can perpetuate stigma towards LGBTI+ people. This neutrality can be interpreted as a lack of support or recognition, which reinforces marginalisation. Recent literature indicates that the absence of active positioning against discrimination amounts to a passive form of stigma perpetuation [58].

Practical training that promotes prejudice-free spaces and provides pleasant environments, such as case analyses that invite reflection and allow for the identification of the importance of improving unfavourable behaviours, is important. Furthermore, it is essential to develop structured curricula that allow theory to be integrated with practice. This could also be implemented through the use of virtual active learning methodologies, which have proven to be as effective as face-to-face active learning methodologies [59]. In the literature, numerous methods used for training have been reported, including presentations, interview sessions, peer learning, group work and round-table discussions with LGBTIQ+ populations. However, in future studies, it would be interesting to evaluate the effectiveness of the educational material in increasing health professions students’ attitudes towards caring for the LGBTIQ+ population, on par with treatment adherence in patients with chronic diseases such as diabetes mellitus, hypertension and cardiovascular disease [60,61].

Bullying is a problem that is found on a large scale in educational environments, a problem that has grown stronger over time and has been little addressed, thus triggering the consolidation of the rejection of historically discriminated groups [62]. Rivera-Osorio and Arias-Gómez [63], in their study conducted in Colombia, identify existing problems that affect young LGTBIQ+ people. This population feels insecure in educational environments (67.0%), receives homophobic comments from most students (25.4%), suffers aggression from adults in the educational institution (37.2%) and experiences a lack of support from school staff (39.9%). LGBTI people are particularly vulnerable to bullying. Larrain Mariño et al. [64] coined the term LGBTI-phobic bullying to refer to ‘harassment motivated by a phobia of the LGBT community, which is underpinned by sexism and values associated with heteronormativity’. On the basis of these values, the victim is subjected to exclusion, isolation, threats, insults and aggression on repeated occasions. The harassment to which the LGBTIQ+ population is subjected can be evidenced in multiple nuances and through different modalities, among which we can highlight being teased, insults, exclusions, physical or verbal aggression and defamation on social networks, as well as other harmful behaviour. Such behaviour can deteriorate mental integrity. Victims of bullying may experience various states of stress, anxiety, depression, self-esteem problems, isolation and suicidal thoughts [65,66,67]. A report delivered by the Office of the Public Defender during a public hearing in Congress on the current policy in favour of the LGBTIQ+ population in Colombia revealed alarming data on violence directed at this population. The Public Defender’s Office received 388 complaints of violence secondary to intolerance towards sexual diversity. Of the total cases reported, 17 were transgender women, who suffered the most serious violence, 12 cases were murders against homosexual men, 6 against lesbian women and 1 case against a transgender man. The region with the highest number of incidents of LGBTI phobia is Caribbean Colombia [68].

Leininger’s theory of the diversity and universality of cultural care emphasises the importance of providing health care that takes into account the beliefs, practices and values of each individual [34,69,70]. Care that is respectful of differences, and in harmony with the beliefs of the individual, promotes comfort, well-being and health outcomes, ensuring a satisfying care experience for the patient and the health professional [71,72]. The results of studies conducted on different populations experiencing discrimination, stigmatisation, bullying or isolation suggest that the implementation of theoretical models of inclusive care, such as the practice of a holistic approach based on empathy and active listening, can improve the relationship between nurses and patients [73,74,75]. Acceptance of sexual and gender diversity in the nursing process ensures effective and barrier-free care. Leininger recognises the importance of beliefs and thoughts; this enables the nurse to develop the human capacity to understand diversity, promoting behaviours that are beneficial to the health and illness process of patients and how their inherent thoughts contribute to their overall state [33,35]. In the education of students, the application of Madeleine Leininger’s transcultural theory could take place, for example, in the following ways:

- Creating case studies in which a non-binary patient experiences prejudice or is reluctant to reveal their sexual identity due to previous discriminatory experiences. In this case, students have to analyse how the nurse can provide more inclusive and welcoming care.
- Including training that addresses how LGBTQ+ people’s history of discrimination and exclusion may influence their experience of care. Students could be trained to recognise signs of anxiety or distress related to past experiences of discrimination and to create a safe environment.
- Instructing student nurses on how to individualise care according to the specific needs and experiences related to gender and sexuality of each patient. When caring for an LGBTQ+ person, the nurse may need to adapt the care plan according to the specific needs of the individual, such as communicating preferred gender, using inclusive language and managing experiences of gender dysphoria, if necessary.

In synthesis, the application of Leininger’s transcultural theory in the education of nurses on sexual and gender diversity not only contributes to enriching professional skills but also promotes a more inclusive and respectful healthcare environment for all patients. Education and awareness-raising on these issues are essential to prepare nursing students for the variety of experiences and cultural identities they will encounter in their professional career.

Despite the results of this study showing that nursing students are interested in improving their training in caring for the LGBTIQ+ population, there are discrepancies in the perception of sexual and gender diversity within the group studied. In particular, some participants showed neutral or negative attitudes, which could be related to the impact of academic training and sociocultural influences that perpetuate the stigmatisation of this population [16,24]. Previous studies have shown that training on cultural competence in sexual diversity not only reduces discrimination but also improves the safety and clinical competence of health professionals [29]. However, other studies warn that the mere incorporation of educational content on sexual diversity in school curricula does not guarantee a transformation of attitudes if these are not accompanied by interactive pedagogical strategies and practical experiences in real care settings for LGBTIQ+ patients [18,31]. In this sense, the adoption of Leininger’s cultural care model can be used to present a key tool to address these contradictions, recognizing that nursing care is based on a holistic application that recognises the value of each patient’s identity and knowledge without prejudice [34,35].

Although this study provides a more detailed insight into the perspective of nursing students in relation to the provision of care for people with sexual and gender diversity, the results must be considered in the light of certain limitations. Firstly, the study was conducted in only one Colombian university; therefore, the results may not be generalisable to other contexts, either in other provinces of Colombia or in other countries, due to potential cultural differences regarding the LGBTIQ+ population. Further studies in other contexts are needed to identify any differences in the perceptions of nursing students in relation to this population. Secondly, the interviewees, although they agreed to participate voluntarily and anonymity was guaranteed, may have felt pressured to give socially desirable answers or talk about topics that are often still taboo today.

## 5. Conclusions

This study found, with regard to the provision of care for people with sexual and gender diversity, that nursing students at the Universidad Popular del Cesar need to comprehensively increase their knowledge. Although some students express the importance of providing inclusive care oriented towards respecting gender diversity, on the other hand, it is noted that there are barriers that limit its provision in clinical practice. Prior theoretical training is important, as well as increasing a gender mainstreaming perspective in order to respond comprehensively to the needs of individuals.

Acceptance of sexual and gender diversity in the nursing education process is essential to ensure the removal of health disparities. It is important to consider the following recommendations: (1) implement education on health and sexual and gender diversity, including activities such as practical training scenarios, workshops and conferences, in order to make visible the specific needs of the LGBTIQ+ population; (2) update health policies and protocols to include specific indications for this population; (3) promote research on the experiences of sexually diverse people in health care environments, analysing advantages and disadvantages in access; (4) promote a differential approach, respecting human dignity and the decisions of others; (5) promote self-care plans to minimize the needs of the LGBTIQ+ community; (6) promote the nursing care process in the clinical context guided by Leininger’s theory; (7) implement topics for curricular content that promotes respect for diversity; (8) pursue diversity-friendly policies to achieve a uniform and holistic care perspective; and, finally, (9) disseminate educational material on sexual and gender diversity and the theory of transcultural care, which calls for accepting differences and making care a transformative process.

Future studies should explore the perceptions of nursing students regarding (a) the importance of specific education on sexual diversity in relation to the quality of care provided; (b) the support of institutions in providing health services to the sexually diverse population during students’ clinical internships—how does the role of the teacher influence the incorporation of inclusive attitudes towards sexual and gender diversity in the future health professional?

## Figures and Tables

**Table 1:** Nursing student perspectives for care in sexually diverse population.

Category	Main Themes Emerged
Care perspective	Personal perspective Professional and disciplinary training Nursing education Health implications

## Data Availability

The data presented in this study are available from the corresponding author upon request.

## Public Involvement Statement

There was no public involvement in any aspect of this research.

## Guidelines and Standards Statement

This manuscript was written following the recommendations of the COREQ guide for qualitative research reporting [49].

## Appendix A. Interview Guide

(1) What is your perception of LGBTIQ+ people?
(2) When you see LGBTI people in marches, parades and protests, what is your reaction?
(3) Do you agree with aggression towards an LGBTI person?
(4) Do you think that an LGBTI person represents a social harm?
(5) What rights do you think are often violated or denied to this population?
(6) Do you agree with sexual diversity?
(7) From your religious point of view, what is your opinion on an LGBTI person?
(8) What do you think about new family models, those different from the traditional family?
(9) Do you think that a person who belongs to the LGBTI community loses values and principles?
(10) Could you decipher the acronym LGBTI?
(11) Do you know the difference between sex and sexual orientation?
(12) Do you think there are laws in Colombia that protect the rights of LGBTI people? If you know, mention at least one.
(13) Do you think that LGBTI people have a mental illness?
(14) What health issues do you think the LGBTI population faces?
(15) Do you know of any protocols or guidelines for caring for LGBTI people, such as inclusive language, treatment, approach or other care tools?
(16) To an LGBTI person who requires care, which nursing theory would you apply to provide holistic care?
(17) Is there a policy at the university that protects the LGBTI population?
(18) Do you think there are barriers to healthcare for LGBTI people? If so, explain your thoughts.
(19) Is there a subject or module in nursing that provides tools for the comprehensive care of LGBTI people using a differential approach?
(20) What do you mean by a differential approach to healthcare?
(21) Have you ever experienced a situation where the right to health of an LGBTI person was violated?
(22) Have you provided care to an LGBTI person in your professional practice?
(23) Do you consider it essential to create a manual with tools for holistic care without stigma and discrimination towards LGBTI people?
